# Poly[μ_2_-aqua-diaqua­(μ_8_-3-nitro­benzene-1,2-dicarboxylato)(μ_6_-3-nitro­benzene-1,2-dicarboxylato)tetra­sodium]

**DOI:** 10.1107/S1600536810044600

**Published:** 2010-11-06

**Authors:** Qi Shuai, You-Ying Di, Ze-Bo Li, Lei Li, Dong-Hua He

**Affiliations:** a College of Science, Northwest A&F University, Yangling 712100, Shanxi Province, People’s Republic of China; b College of Chemistry and Engineering, Liaocheng University, Shandong 252059, People’s Republic of China

## Abstract

In the title layered coordination polymer, [Na_4_(C_8_H_3_NO_6_)_2_(H_2_O)_3_]_*n*_, the doubly deprotonated 3-nitro­benzene-1,2-dicarboxyl­ate ligands exhibit μ_8_- and μ_6_-coordination modes to the sodium ions, generating sheets lying parallel to (001). The coordination environments of the sodium ions are distorted octa­hedral, distorted trigonal-bipyramidal and moncapped trigonal-prismatic. One of the nitro groups is disordered over two sets of sites with site-occupancy factors 0.580 (8):0.419 (2). A network of O—H⋯O and O—H⋯N hydrogen bonds helps to establish the packing.

## Related literature

For a related structure containing the same components, see: Guo (2004 ▶). 
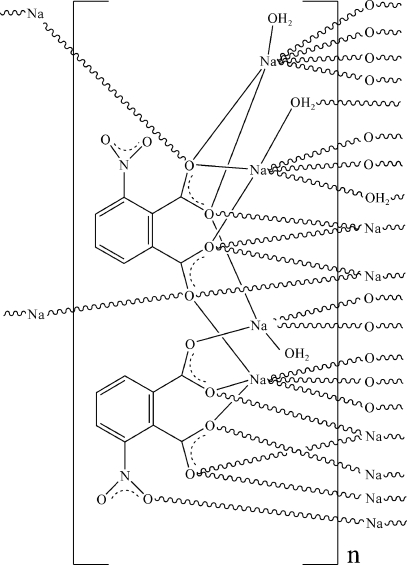


## Experimental

### 

#### Crystal data

[Na_4_(C_8_H_3_NO_6_)_2_(H_2_O)_3_]
*M* *_r_* = 564.24
Triclinic, 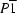

*a* = 6.6871 (8) Å
*b* = 10.6193 (15) Å
*c* = 14.582 (2) Å
α = 82.065 (1)°
β = 83.428 (1)°
γ = 89.371 (2)°
*V* = 1018.8 (2) Å^3^
*Z* = 2
Mo *K*α radiation
μ = 0.23 mm^−1^
*T* = 298 K
0.44 × 0.38 × 0.17 mm

#### Data collection

Bruker SMART CCD diffractometer
Absorption correction: multi-scan (*SADABS*; Bruker, 2002 ▶) *T*_min_ = 0.905, *T*_max_ = 0.962
5276 measured reflections
3514 independent reflections
2272 reflections with *I* > 2σ(*I*)
*R*_int_ = 0.026

#### Refinement

*R*[*F* ^2^ > 2σ(*F* ^2^)] = 0.065
*wR*(*F* ^2^) = 0.208
*S* = 1.04
3514 reflections
344 parameters
162 restraints
H-atom parameters constrained
Δρ_max_ = 0.76 e Å^−3^
Δρ_min_ = −0.69 e Å^−3^

### 

Data collection: *SMART* (Bruker, 2002 ▶); cell refinement: *SAINT* (Bruker, 2002 ▶); data reduction: *SAINT*; program(s) used to solve structure: *SHELXS97* (Sheldrick, 2008 ▶); program(s) used to refine structure: *SHELXL97* (Sheldrick, 2008 ▶); molecular graphics: *SHELXTL* (Sheldrick, 2008 ▶); software used to prepare material for publication: *SHELXL97*.

## Supplementary Material

Crystal structure: contains datablocks global, I. DOI: 10.1107/S1600536810044600/hb5692sup1.cif

Structure factors: contains datablocks I. DOI: 10.1107/S1600536810044600/hb5692Isup2.hkl

Additional supplementary materials:  crystallographic information; 3D view; checkCIF report

## Figures and Tables

**Table 1:** Selected bond lengths (Å)

Na1—O2	2.323 (4)
Na1—O8^i^	2.354 (4)
Na1—O2^ii^	2.357 (4)
Na1—O8	2.374 (4)
Na1—O10	2.502 (5)
Na1—O9^i^	2.511 (5)
Na2—O1^iii^	2.312 (4)
Na2—O1	2.320 (4)
Na2—O13	2.326 (4)
Na2—O13^iv^	2.353 (4)
Na2—O3^iii^	2.474 (4)
Na2—O4	2.475 (4)
Na3—O7	2.270 (4)
Na3—O3	2.358 (4)
Na3—O4^v^	2.390 (4)
Na3—O14	2.419 (7)
Na3—O11^vi^	2.488 (5)
Na4—O10^vi^	2.363 (5)
Na4—O1^iii^	2.437 (4)
Na4—O15	2.448 (6)
Na4—O3	2.546 (4)
Na4—O2^iii^	2.563 (4)
Na4—O4	2.647 (4)
Na4—O9^vii^	2.755 (6)

Symmetry codes: (i) 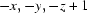; (ii) 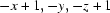; (iii) 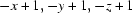; (iv) 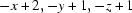; (v) 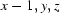; (vi) 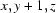; (vii) 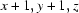.

**Table 2:** Hydrogen-bond geometry (Å, °)

*D*—H⋯*A*	*D*—H	H⋯*A*	*D*⋯*A*	*D*—H⋯*A*
O13—H13*A*⋯O7^iii^	0.85	1.94	2.789 (6)	180
O13—H13*A*⋯O8^iii^	0.85	2.51	3.049 (5)	123
O13—H13*B*⋯O10^vii^	0.85	2.13	2.980 (6)	180
O13—H13*B*⋯O9^vii^	0.85	2.63	3.186 (6)	125
O14—H14*B*⋯O6^viii^	0.85	1.93	2.782 (13)	179
O14—H14*B*⋯O6′^viii^	0.85	1.97	2.722 (15)	147
O14—H14*C*⋯O6′^v^	0.85	1.45	2.29 (2)	166
O14—H14*C*⋯N1^v^	0.85	2.27	3.078 (10)	160
O14—H14*C*⋯O6^v^	0.85	2.55	3.388 (18)	171
O15—H15*B*⋯O5	0.85	2.13	2.951 (7)	162
O15—H15*C*⋯O9^vii^	0.85	2.29	2.782 (8)	117
O15—H15*C*⋯O11^vii^	0.85	2.48	3.302 (8)	163

Symmetry codes: (iii) 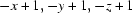; (v) 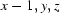; (vii) 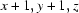; (viii) 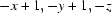.

**Fig. 1.**
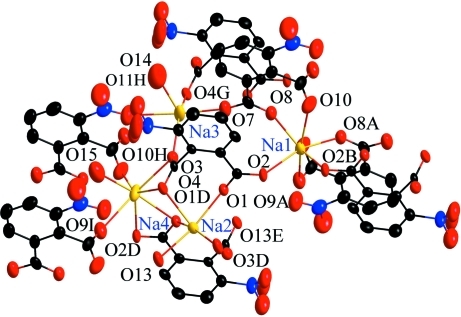
Coordination environment of NaI ions in the title complex. Non-hydrogen atoms are shown as 30% probability ellipsoids. Hydrogen atoms are omitted for clarity. Symmetry codes: (A) -x, -y, -z + 1; (B) -x + 1, -y, -z + 1; (D) -x + 1, -y + 1, -z + 1; (E) -x + 2, -y + 1, -z + 1; (G) x - 1, y, z; (H) x, y + 1, z; (I) x + 1, y + 1, z.

**Fig. 2.**
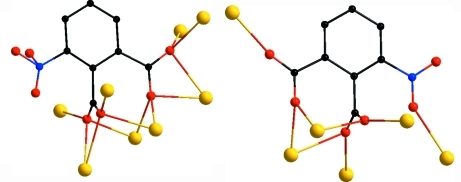
Coordination modes of 3-Nitrobenzene-1,2-dicarboxylic acid ligands in the title complex. Hydrogen atoms are omitted for clarity.

**Fig. 3.**
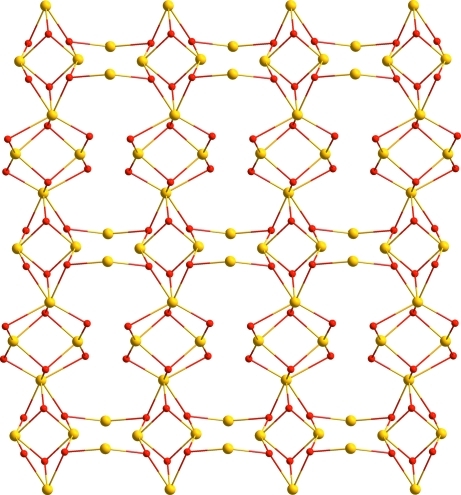
View of two-dimensional framework along c axis in the title complex.

## supplementary crystallographic information

### Comment

Ming Ling Guo has obtained one kind of sodium complex {[Na(C_8_H_4_NO_6_)(H_2_O)_3_].H_2_O}_n_ (Guo, 2004) based on 3-Nitrobenzene-1,2-dicarboxylic acid ligand.

Here we report another kind of sodium complex (I) based on the same ligand. Different from {[Na(C_8_H_4_NO_6_)(H_2_O)_3_].H_2_O}_n_, the formula for the title complex is [Na_4_(C_8_H_3_NO_6_)_2_(H_2_O)_3_]_n_. X-ray single-crystal diffraction analysis indicates the presence of four independent Na^I^ ions, two 3-Nitrobenzene-1,2-dicarboxylato and three coordinated water molecules in the asymmetric unit. Only one independent Na^I^ ion can be found in complex of {[Na(C_8_H_4_NO_6_)(H_2_O)_3_].H_2_O}_n_. Moreover, the ligand in the title complex is completely deprotonated, which is different from the uncomplete form in complex of {[Na(C_8_H_4_NO_6_)(H_2_O)_3_].H_2_O}_n_.

In the title complex, the coordination geometry (Fig. 1) around Na1^I^ and Na2^I^ ions could be described as distorted octahedral arrangements with coordination number of 6, all the coordinated atoms are oxygen atoms. For Na1^I^ center, all the six oxygen atoms come from carboxylate groups. For Na2^I^ center, four oxygen atoms come from carboxylate groups, two of them come from coordinated water molecules. The coordination numbers of Na3^I^ and Na4^I^ ions are 5 and 7, and the geometries around them could be described as distorted trigonal bipyramid and moncapped octahedron prism arrangements. Around Na3^I^ center, three oxygen atoms come from carboxylate groups, one is nitro oxygen atom, and the last comes from coordinated water molecule. Concerning Na4^I^ center, six of the oxygen atoms belong to carboxylate groups and only one oxygen atom is from coordinated water molecule. 3-Nitrobenzene-1,2-dicarboxylic acid ligands exhibit two coordination modes (Fig. 2), which can be classified as µ_8_-(κ^12^, O^1^: O^1^: O^1^: O^2^: O^2^: O^2^: O^3^: O^3^: O^3^: O^4^: O^4^: O^4^) and µ_6_-(κ^8^, O^7^: O^8^: O^8^: O^9^: O^9^: O^10^: O^10^: O^11^), and a two-dimensional (Fig. 3) polymeric framwork can be assembled by oxygen atoms of the ligands through the two coordination modes. We can clearly see that it is different from the complex synthesized by Ming Ling Guo, which forms polymeric chains by way of edge-sharing *via* pairs of water molecules between NaO(H_2_O)_5_ octahedra.

### Experimental

A mixture of strontium chloride hexahydrate (0.0267 g, 0.1 mmol), sodium hydroxide (0.0080 g, 0.2 mmol), 3-Nitrobenzene-1,2-dicarboxylic acid (0.0211 g, 0.1 mmol), and H_2_O (20 mL) was placed in a Parr Teflon-lined stainless stell vessel (25 ml), and then the vessel was sealed and heated at 443.15 K for 4 days. Then the vessel was cooled to 373.15 K at a rate of 5 K h^-1^ and slowly cooled to room temperature. Colorless, block single crystals suitable for X-ray diffraction were obtained.

### Crystal data

**Table:**

[Na_4_(C_8_H_3_NO_6_)_2_(H_2_O)_3_]	*Z* = 2
*M**_r_* = 564.24	*F*(000) = 572
Triclinic, *P*1	*D*_x_ = 1.839 Mg m^−^^3^
Hall symbol: -P 1	Mo *K*α radiation, λ = 0.71073 Å
*a* = 6.6871 (8) Å	Cell parameters from 1631 reflections
*b* = 10.6193 (15) Å	θ = 2.8–28.2°
*c* = 14.582 (2) Å	µ = 0.23 mm^−^^1^
α = 82.065 (1)°	*T* = 298 K
β = 83.428 (1)°	Block, colourless
γ = 89.371 (2)°	0.44 × 0.38 × 0.17 mm
*V* = 1018.8 (2) Å^3^	

### Data collection

**Table:**

Bruker SMART CCD diffractometer	3514 independent reflections
Radiation source: fine-focus sealed tube	2272 reflections with *I* > 2σ(*I*)
graphite	*R*_int_ = 0.026
phi and ω scans	θ_max_ = 25.0°, θ_min_ = 2.8°
Absorption correction: multi-scan (*SADABS*; Bruker, 2002)	*h* = −7→7
*T*_min_ = 0.905, *T*_max_ = 0.962	*k* = −11→12
5276 measured reflections	*l* = −17→14

### Refinement

**Table:**

Refinement on *F*^2^	Primary atom site location: structure-invariant direct methods
Least-squares matrix: full	Secondary atom site location: difference Fourier map
*R*[*F*^2^ > 2σ(*F*^2^)] = 0.065	Hydrogen site location: inferred from neighbouring sites
*wR*(*F*^2^) = 0.208	H-atom parameters constrained
*S* = 1.04	*w* = 1/[σ^2^(*F*_o_^2^) + (0.1154*P*)^2^ + 0.9421*P*] where *P* = (*F*_o_^2^ + 2*F*_c_^2^)/3
3514 reflections	(Δ/σ)_max_ < 0.001
344 parameters	Δρ_max_ = 0.76 e Å^−^^3^
162 restraints	Δρ_min_ = −0.69 e Å^−^^3^

### Special details

**Table:**

Geometry. All e.s.d.'s (except the e.s.d. in the dihedral angle between two l.s. planes) are estimated using the full covariance matrix. The cell e.s.d.'s are taken into account individually in the estimation of e.s.d.'s in distances, angles and torsion angles; correlations between e.s.d.'s in cell parameters are only used when they are defined by crystal symmetry. An approximate (isotropic) treatment of cell e.s.d.'s is used for estimating e.s.d.'s involving l.s. planes.
Refinement. Refinement of *F*^2^ against ALL reflections. The weighted *R*-factor *wR* and goodness of fit *S* are based on *F*^2^, conventional *R*-factors *R* are based on *F*, with *F* set to zero for negative *F*^2^. The threshold expression of *F*^2^ > σ(*F*^2^) is used only for calculating *R*-factors(gt) *etc*. and is not relevant to the choice of reflections for refinement. *R*-factors based on *F*^2^ are statistically about twice as large as those based on *F*, and *R*- factors based on ALL data will be even larger.

### Fractional atomic coordinates and isotropic or equivalent isotropic displacement parameters (Å^2^)

**Table:**

	*x*	*y*	*z*	*U*_iso_*/*U*_eq_	Occ. (<1)
Na1	0.2359 (3)	0.00241 (17)	0.50423 (14)	0.0330 (5)	
Na2	0.7386 (3)	0.50445 (17)	0.49820 (13)	0.0302 (5)	
Na3	0.0578 (3)	0.5315 (2)	0.28461 (17)	0.0479 (6)	
Na4	0.5016 (4)	0.7672 (2)	0.37516 (15)	0.0522 (7)	
N1	0.6311 (9)	0.5233 (5)	0.1143 (3)	0.0533 (14)	
N2	0.1544 (9)	−0.1646 (5)	0.1478 (4)	0.0556 (14)	
O1	0.5149 (5)	0.3531 (3)	0.4692 (2)	0.0299 (8)	
O2	0.5038 (5)	0.1449 (3)	0.4694 (2)	0.0335 (8)	
O3	0.3758 (5)	0.5587 (3)	0.3345 (2)	0.0328 (8)	
O4	0.7092 (5)	0.5666 (3)	0.3304 (2)	0.0324 (8)	
O5	0.5745 (9)	0.6168 (4)	0.1406 (3)	0.0703 (13)	
O6	0.645 (2)	0.5248 (9)	0.0290 (6)	0.084 (3)	0.581 (14)
O6'	0.794 (3)	0.5271 (13)	0.0538 (10)	0.085 (4)	0.419 (14)
O7	0.0464 (6)	0.3179 (4)	0.3283 (3)	0.0511 (10)	
O8	0.0225 (6)	0.1255 (3)	0.4077 (3)	0.0434 (9)	
O9	−0.1331 (8)	−0.1086 (4)	0.3542 (3)	0.0583 (11)	
O10	0.1955 (8)	−0.1197 (4)	0.3725 (3)	0.0644 (11)	
O11	0.0978 (9)	−0.2416 (5)	0.2146 (3)	0.0735 (13)	
O12	0.2147 (11)	−0.1972 (6)	0.0737 (4)	0.104 (2)	
O13	1.0048 (6)	0.6475 (3)	0.4843 (3)	0.0438 (10)	
H13A	0.9897	0.6583	0.5414	0.053*	
H13B	1.0595	0.7139	0.4526	0.053*	
O14	0.0893 (12)	0.5436 (6)	0.1167 (5)	0.116 (2)	
H14B	0.1713	0.5230	0.0724	0.139*	
H14C	−0.0290	0.5412	0.1009	0.139*	
O15	0.6276 (9)	0.8569 (5)	0.2156 (4)	0.0861 (16)	
H15B	0.5852	0.7932	0.1939	0.103*	
H15C	0.7525	0.8465	0.2201	0.103*	
C1	0.5209 (7)	0.2562 (4)	0.4276 (3)	0.0250 (10)	
C2	0.5488 (7)	0.5173 (4)	0.3145 (3)	0.0235 (10)	
C3	0.5542 (7)	0.2752 (4)	0.3225 (3)	0.0256 (11)	
C4	0.5689 (7)	0.3952 (4)	0.2700 (3)	0.0224 (10)	
C5	0.6096 (8)	0.4004 (5)	0.1743 (3)	0.0328 (12)	
C6	0.6326 (9)	0.2933 (5)	0.1290 (4)	0.0418 (14)	
H6	0.6595	0.3009	0.0645	0.050*	
C7	0.6145 (8)	0.1769 (5)	0.1820 (4)	0.0384 (13)	
H7	0.6276	0.1036	0.1535	0.046*	
C8	0.5770 (8)	0.1676 (5)	0.2771 (4)	0.0320 (12)	
H8	0.5664	0.0875	0.3124	0.038*	
C9	0.0500 (8)	0.1990 (5)	0.3334 (4)	0.0368 (13)	
C10	0.0487 (11)	−0.0812 (5)	0.3305 (4)	0.0437 (15)	
C11	0.0931 (8)	0.1445 (5)	0.2430 (4)	0.0379 (13)	
C12	0.0976 (8)	0.0117 (5)	0.2412 (4)	0.0376 (13)	
C13	0.1464 (9)	−0.0290 (6)	0.1550 (4)	0.0448 (14)	
C14	0.1874 (10)	0.0541 (7)	0.0734 (4)	0.0580 (18)	
H14A	0.2219	0.0232	0.0171	0.070*	
C15	0.1767 (11)	0.1815 (7)	0.0762 (5)	0.0615 (19)	
H15A	0.2003	0.2383	0.0215	0.074*	
C16	0.1308 (9)	0.2257 (6)	0.1606 (5)	0.0510 (16)	
H16	0.1252	0.3129	0.1621	0.061*	

### Atomic displacement parameters (Å^2^)

**Table:**

	*U*^11^	*U*^22^	*U*^33^	*U*^12^	*U*^13^	*U*^23^
Na1	0.0312 (11)	0.0249 (11)	0.0412 (12)	−0.0031 (8)	−0.0024 (9)	0.0004 (9)
Na2	0.0293 (11)	0.0276 (11)	0.0333 (11)	−0.0011 (8)	−0.0008 (8)	−0.0051 (8)
Na3	0.0333 (13)	0.0374 (13)	0.0668 (16)	−0.0031 (10)	−0.0023 (11)	0.0127 (11)
Na4	0.0914 (19)	0.0293 (12)	0.0323 (12)	0.0099 (12)	0.0022 (12)	0.0002 (9)
N1	0.092 (4)	0.037 (3)	0.026 (3)	−0.010 (3)	0.010 (3)	−0.001 (2)
N2	0.070 (4)	0.059 (4)	0.038 (3)	0.009 (3)	−0.004 (3)	−0.008 (3)
O1	0.0361 (18)	0.0217 (16)	0.0308 (16)	−0.0013 (13)	−0.0004 (14)	−0.0032 (13)
O2	0.0379 (19)	0.0186 (16)	0.0406 (19)	−0.0014 (14)	−0.0009 (15)	0.0050 (14)
O3	0.0352 (18)	0.0261 (16)	0.0368 (18)	0.0034 (14)	−0.0020 (14)	−0.0061 (14)
O4	0.0358 (18)	0.0217 (16)	0.0388 (18)	−0.0050 (14)	0.0000 (14)	−0.0046 (13)
O5	0.108 (3)	0.043 (2)	0.052 (2)	0.008 (2)	0.010 (2)	0.004 (2)
O6	0.135 (7)	0.063 (5)	0.047 (5)	−0.008 (5)	0.009 (5)	−0.002 (4)
O6'	0.110 (8)	0.061 (6)	0.065 (6)	0.008 (6)	0.040 (6)	0.014 (5)
O7	0.052 (2)	0.034 (2)	0.064 (2)	0.0012 (17)	−0.0030 (19)	0.0017 (17)
O8	0.052 (2)	0.0327 (18)	0.0437 (19)	−0.0015 (16)	−0.0045 (16)	0.0015 (16)
O9	0.082 (3)	0.041 (2)	0.048 (2)	−0.010 (2)	0.006 (2)	−0.0049 (17)
O10	0.090 (3)	0.051 (2)	0.049 (2)	0.024 (2)	−0.009 (2)	−0.0013 (18)
O11	0.114 (3)	0.049 (2)	0.053 (2)	0.011 (2)	0.008 (2)	−0.004 (2)
O12	0.171 (5)	0.081 (4)	0.057 (3)	0.001 (4)	0.020 (3)	−0.025 (3)
O13	0.042 (2)	0.0249 (18)	0.062 (2)	−0.0068 (16)	0.0022 (18)	−0.0012 (17)
O14	0.143 (5)	0.094 (4)	0.099 (4)	0.000 (4)	0.020 (4)	−0.002 (3)
O15	0.088 (4)	0.057 (3)	0.111 (4)	−0.003 (3)	−0.002 (3)	−0.008 (3)
C1	0.020 (2)	0.024 (3)	0.031 (3)	0.0011 (19)	−0.0023 (19)	−0.002 (2)
C2	0.029 (3)	0.015 (2)	0.025 (2)	−0.004 (2)	0.002 (2)	−0.0002 (18)
C3	0.019 (2)	0.028 (3)	0.030 (3)	−0.001 (2)	−0.0028 (19)	−0.004 (2)
C4	0.021 (2)	0.022 (2)	0.024 (2)	−0.0027 (19)	0.0006 (19)	−0.0054 (19)
C5	0.038 (3)	0.029 (3)	0.030 (3)	−0.004 (2)	0.001 (2)	−0.003 (2)
C6	0.056 (4)	0.042 (3)	0.028 (3)	0.001 (3)	0.002 (3)	−0.011 (2)
C7	0.043 (3)	0.036 (3)	0.039 (3)	−0.002 (2)	−0.003 (3)	−0.018 (3)
C8	0.031 (3)	0.023 (3)	0.044 (3)	0.000 (2)	−0.007 (2)	−0.011 (2)
C9	0.024 (3)	0.030 (3)	0.057 (4)	−0.004 (2)	−0.006 (2)	−0.003 (3)
C10	0.069 (4)	0.029 (3)	0.033 (3)	0.014 (3)	−0.008 (3)	−0.004 (2)
C11	0.026 (3)	0.035 (3)	0.048 (3)	−0.004 (2)	−0.003 (2)	0.009 (2)
C12	0.031 (3)	0.039 (3)	0.041 (3)	0.001 (2)	−0.005 (2)	0.003 (2)
C13	0.047 (4)	0.047 (4)	0.039 (3)	0.000 (3)	−0.005 (3)	0.001 (3)
C14	0.061 (4)	0.075 (5)	0.035 (4)	−0.008 (4)	−0.005 (3)	0.004 (3)
C15	0.064 (5)	0.063 (5)	0.050 (4)	−0.018 (4)	−0.007 (3)	0.020 (3)
C16	0.044 (4)	0.045 (4)	0.059 (4)	−0.010 (3)	−0.007 (3)	0.011 (3)

### Geometric parameters (Å, °)

**Table:**

Na1—O2	2.323 (4)	O4—Na3^viii^	2.390 (4)
Na1—O8^i^	2.354 (4)	O7—C9	1.255 (6)
Na1—O2^ii^	2.357 (4)	O8—C9	1.240 (7)
Na1—O8	2.374 (4)	O8—Na1^i^	2.354 (4)
Na1—O10	2.502 (5)	O9—C10	1.252 (8)
Na1—O9^i^	2.511 (5)	O9—Na1^i^	2.511 (5)
Na2—O1^iii^	2.312 (4)	O9—Na4^ix^	2.755 (6)
Na2—O1	2.320 (4)	O10—C10	1.251 (7)
Na2—O13	2.326 (4)	O10—Na4^x^	2.363 (5)
Na2—O13^iv^	2.353 (4)	O11—Na3^x^	2.488 (5)
Na2—O3^iii^	2.474 (4)	O13—Na2^iv^	2.353 (4)
Na2—O4	2.475 (4)	O13—H13A	0.8498
Na3—O7	2.270 (4)	O13—H13B	0.8500
Na3—O3	2.358 (4)	O14—H14B	0.8500
Na3—O4^v^	2.390 (4)	O14—H14C	0.8500
Na3—O14	2.419 (7)	O15—H15B	0.8500
Na3—O11^vi^	2.488 (5)	O15—H15C	0.8500
Na4—O10^vi^	2.363 (5)	C1—C3	1.509 (7)
Na4—O1^iii^	2.437 (4)	C2—C4	1.526 (6)
Na4—O15	2.448 (6)	C3—C4	1.391 (7)
Na4—O3	2.546 (4)	C3—C8	1.396 (7)
Na4—O2^iii^	2.563 (4)	C4—C5	1.385 (7)
Na4—O4	2.647 (4)	C5—C6	1.390 (7)
Na4—O9^vii^	2.755 (6)	C6—C7	1.363 (8)
N1—O5	1.156 (6)	C6—H6	0.9300
N1—O6	1.236 (10)	C7—C8	1.371 (7)
N1—O6'	1.320 (15)	C7—H7	0.9300
N1—C5	1.466 (7)	C8—H8	0.9300
N2—O12	1.204 (7)	C9—C11	1.509 (8)
N2—O11	1.209 (7)	C10—C12	1.527 (8)
N2—C13	1.458 (8)	C11—C16	1.378 (8)
O1—C1	1.263 (6)	C11—C12	1.414 (8)
O1—Na2^iii^	2.312 (4)	C12—C13	1.387 (8)
O1—Na4^iii^	2.437 (4)	C13—C14	1.382 (8)
O2—C1	1.253 (6)	C14—C15	1.359 (10)
O2—Na1^ii^	2.357 (4)	C14—H14A	0.9300
O2—Na4^iii^	2.563 (4)	C15—C16	1.379 (10)
O3—C2	1.250 (6)	C15—H15A	0.9300
O3—Na2^iii^	2.474 (4)	C16—H16	0.9300
O4—C2	1.257 (6)		
			
O2—Na1—O8^i^	159.75 (16)	C2—O4—Na2	106.5 (3)
O2—Na1—O2^ii^	82.43 (13)	Na3^viii^—O4—Na2	93.63 (14)
O8^i^—Na1—O2^ii^	95.74 (14)	C2—O4—Na4	89.2 (3)
O2—Na1—O8	94.71 (14)	Na3^viii^—O4—Na4	133.50 (16)
O8^i^—Na1—O8	95.73 (14)	Na2—O4—Na4	88.06 (12)
O2^ii^—Na1—O8	153.10 (16)	C9—O7—Na3	167.2 (4)
O2—Na1—O10	112.15 (17)	C9—O8—Na1^i^	139.0 (4)
O8^i^—Na1—O10	87.46 (16)	C9—O8—Na1	134.7 (4)
O2^ii^—Na1—O10	82.65 (15)	Na1^i^—O8—Na1	84.27 (14)
O8—Na1—O10	73.64 (15)	C10—O9—Na1^i^	105.9 (4)
O2—Na1—O9^i^	87.49 (15)	C10—O9—Na4^ix^	161.6 (4)
O8^i^—Na1—O9^i^	74.31 (15)	Na1^i^—O9—Na4^ix^	91.05 (16)
O2^ii^—Na1—O9^i^	111.21 (16)	C10—O10—Na4^x^	149.5 (4)
O8—Na1—O9^i^	95.30 (16)	C10—O10—Na1	111.7 (4)
O10—Na1—O9^i^	157.80 (19)	Na4^x^—O10—Na1	97.44 (19)
O1^iii^—Na2—O1	93.04 (13)	N2—O11—Na3^x^	148.5 (4)
O1^iii^—Na2—O13	96.65 (14)	Na2—O13—Na2^iv^	96.95 (14)
O1—Na2—O13	163.33 (16)	Na2—O13—H13A	94.9
O1^iii^—Na2—O13^iv^	162.13 (16)	Na2^iv^—O13—H13A	94.7
O1—Na2—O13^iv^	91.84 (14)	Na2—O13—H13B	144.8
O13—Na2—O13^iv^	83.05 (14)	Na2^iv^—O13—H13B	106.7
O1^iii^—Na2—O3^iii^	75.90 (13)	H13A—O13—H13B	108.4
O1—Na2—O3^iii^	86.70 (13)	Na3—O14—H14B	139.6
O13—Na2—O3^iii^	108.79 (15)	Na3—O14—H14C	107.4
O13^iv^—Na2—O3^iii^	87.24 (14)	H14B—O14—H14C	108.1
O1^iii^—Na2—O4	88.52 (13)	Na4—O15—H15B	91.7
O1—Na2—O4	78.00 (12)	Na4—O15—H15C	97.8
O13—Na2—O4	88.70 (14)	H15B—O15—H15C	107.9
O13^iv^—Na2—O4	109.32 (14)	O2—C1—O1	123.1 (4)
O3^iii^—Na2—O4	157.58 (14)	O2—C1—C3	118.4 (4)
O7—Na3—O3	94.91 (15)	O1—C1—C3	118.4 (4)
O7—Na3—O4^v^	95.67 (15)	O2—C1—Na4^iii^	64.5 (3)
O3—Na3—O4^v^	140.24 (16)	O1—C1—Na4^iii^	58.8 (2)
O7—Na3—O14	101.1 (2)	C3—C1—Na4^iii^	174.0 (3)
O3—Na3—O14	110.0 (2)	O3—C2—O4	125.2 (4)
O4^v^—Na3—O14	105.3 (2)	O3—C2—C4	118.1 (4)
O7—Na3—O11^vi^	171.5 (2)	O4—C2—C4	116.7 (4)
O3—Na3—O11^vi^	84.59 (17)	O3—C2—Na4	60.6 (2)
O4^v^—Na3—O11^vi^	90.07 (16)	O4—C2—Na4	65.3 (2)
O14—Na3—O11^vi^	71.2 (2)	C4—C2—Na4	172.4 (3)
O10^vi^—Na4—O1^iii^	105.27 (16)	O3—C2—Na2	103.5 (3)
O10^vi^—Na4—O15	93.48 (18)	O4—C2—Na2	50.4 (2)
O1^iii^—Na4—O15	161.21 (19)	C4—C2—Na2	114.3 (3)
O10^vi^—Na4—O3	97.84 (18)	Na4—C2—Na2	72.89 (12)
O1^iii^—Na4—O3	82.68 (13)	C4—C3—C8	119.3 (4)
O15—Na4—O3	96.15 (17)	C4—C3—C1	122.5 (4)
O10^vi^—Na4—O2^iii^	81.22 (15)	C8—C3—C1	118.2 (4)
O1^iii^—Na4—O2^iii^	52.44 (11)	C5—C4—C3	117.1 (4)
O15—Na4—O2^iii^	131.77 (18)	C5—C4—C2	120.5 (4)
O3—Na4—O2^iii^	132.08 (14)	C3—C4—C2	122.4 (4)
O10^vi^—Na4—O4	147.23 (18)	C4—C5—C6	123.7 (5)
O1^iii^—Na4—O4	82.10 (12)	C4—C5—N1	120.4 (4)
O15—Na4—O4	82.72 (16)	C6—C5—N1	116.0 (5)
O3—Na4—O4	50.71 (12)	C7—C6—C5	118.0 (5)
O2^iii^—Na4—O4	124.99 (14)	C7—C6—H6	121.0
O10^vi^—Na4—O9^vii^	121.48 (18)	C5—C6—H6	121.0
O1^iii^—Na4—O9^vii^	103.59 (15)	C6—C7—C8	120.2 (5)
O15—Na4—O9^vii^	64.33 (17)	C6—C7—H7	119.9
O3—Na4—O9^vii^	135.66 (16)	C8—C7—H7	119.9
O2^iii^—Na4—O9^vii^	77.88 (14)	C7—C8—C3	121.7 (5)
O4—Na4—O9^vii^	86.19 (14)	C7—C8—H8	119.1
O5—N1—O6	115.1 (7)	C3—C8—H8	119.1
O5—N1—O6'	118.7 (8)	O8—C9—O7	123.9 (5)
O6—N1—O6'	51.1 (8)	O8—C9—C11	119.2 (5)
O5—N1—C5	121.9 (5)	O7—C9—C11	116.9 (5)
O6—N1—C5	118.6 (6)	O10—C10—O9	127.4 (6)
O6'—N1—C5	111.5 (7)	O10—C10—C12	116.0 (6)
O12—N2—O11	121.3 (6)	O9—C10—C12	116.6 (5)
O12—N2—C13	118.4 (6)	O10—C10—Na1^i^	99.6 (4)
O11—N2—C13	120.3 (5)	O9—C10—Na1^i^	51.2 (3)
C1—O1—Na2^iii^	132.4 (3)	C12—C10—Na1^i^	120.5 (3)
C1—O1—Na2	137.6 (3)	C16—C11—C12	119.3 (6)
Na2^iii^—O1—Na2	86.96 (13)	C16—C11—C9	119.4 (5)
C1—O1—Na4^iii^	94.9 (3)	C12—C11—C9	121.3 (5)
Na2^iii^—O1—Na4^iii^	97.15 (14)	C13—C12—C11	116.9 (5)
Na2—O1—Na4^iii^	94.76 (14)	C13—C12—C10	122.3 (5)
C1—O2—Na1	133.0 (3)	C11—C12—C10	120.8 (5)
C1—O2—Na1^ii^	127.2 (3)	C14—C13—C12	122.8 (6)
Na1—O2—Na1^ii^	97.57 (13)	C14—C13—N2	117.2 (6)
C1—O2—Na4^iii^	89.3 (3)	C12—C13—N2	119.9 (5)
Na1—O2—Na4^iii^	100.56 (15)	C15—C14—C13	119.4 (6)
Na1^ii^—O2—Na4^iii^	95.93 (14)	C15—C14—H14A	120.3
C2—O3—Na3	135.0 (3)	C13—C14—H14A	120.3
C2—O3—Na2^iii^	110.4 (3)	C14—C15—C16	119.5 (6)
Na3—O3—Na2^iii^	94.88 (14)	C14—C15—H15A	120.3
C2—O3—Na4	94.0 (3)	C16—C15—H15A	120.3
Na3—O3—Na4	124.18 (16)	C11—C16—C15	122.0 (6)
Na2^iii^—O3—Na4	88.46 (12)	C11—C16—H16	119.0
C2—O4—Na3^viii^	133.8 (3)	C15—C16—H16	119.0
			
O1^iii^—Na2—O1—C1	160.4 (5)	Na1^ii^—O2—C1—C3	77.2 (5)
O13—Na2—O1—C1	34.9 (8)	Na4^iii^—O2—C1—C3	174.1 (4)
O13^iv^—Na2—O1—C1	−36.8 (5)	Na2^iii^—O1—C1—O2	−99.4 (5)
O3^iii^—Na2—O1—C1	−123.9 (5)	Na2—O1—C1—O2	107.5 (5)
O4—Na2—O1—C1	72.6 (5)	Na4^iii^—O1—C1—O2	5.0 (5)
C2—Na2—O1—C1	81.8 (5)	Na2^iii^—O1—C1—C3	81.9 (5)
O1^iii^—Na2—O1—Na2^iii^	0.0	Na2—O1—C1—C3	−71.3 (6)
O13—Na2—O1—Na2^iii^	−125.5 (5)	Na4^iii^—O1—C1—C3	−173.8 (4)
O13^iv^—Na2—O1—Na2^iii^	162.80 (15)	Na3—O3—C2—O4	160.6 (3)
O3^iii^—Na2—O1—Na2^iii^	75.68 (12)	Na2^iii^—O3—C2—O4	−79.4 (5)
O4—Na2—O1—Na2^iii^	−87.84 (12)	Na4—O3—C2—O4	10.4 (5)
C2—Na2—O1—Na2^iii^	−78.63 (14)	Na3—O3—C2—C4	−21.3 (6)
O1^iii^—Na2—O1—Na4^iii^	−96.93 (14)	Na2^iii^—O3—C2—C4	98.7 (4)
O13—Na2—O1—Na4^iii^	137.5 (5)	Na4—O3—C2—C4	−171.5 (4)
O13^iv^—Na2—O1—Na4^iii^	65.87 (15)	Na3^viii^—O4—C2—O3	−170.1 (3)
O3^iii^—Na2—O1—Na4^iii^	−21.25 (13)	Na2—O4—C2—O3	77.8 (5)
O4—Na2—O1—Na4^iii^	175.23 (14)	Na4—O4—C2—O3	−10.0 (5)
C2—Na2—O1—Na4^iii^	−175.56 (18)	Na3^viii^—O4—C2—C4	11.7 (6)
O8^i^—Na1—O2—C1	−110.5 (6)	Na2—O4—C2—C4	−100.4 (4)
O2^ii^—Na1—O2—C1	163.4 (5)	Na4—O4—C2—C4	171.9 (4)
O8—Na1—O2—C1	10.3 (5)	O10^vi^—Na4—C2—O3	−18.2 (3)
O10—Na1—O2—C1	84.6 (5)	O1^iii^—Na4—C2—O3	86.0 (3)
O9^i^—Na1—O2—C1	−84.8 (5)	O15—Na4—C2—O3	−110.6 (3)
O8^i^—Na1—O2—Na1^ii^	86.1 (4)	O2^iii^—Na4—C2—O3	94.5 (3)
O2^ii^—Na1—O2—Na1^ii^	0.0	O4—Na4—C2—O3	170.6 (4)
O8—Na1—O2—Na1^ii^	−153.09 (16)	O9^vii^—Na4—C2—O3	−171.8 (3)
O10—Na1—O2—Na1^ii^	−78.85 (17)	C1^iii^—Na4—C2—O3	88.3 (3)
O9^i^—Na1—O2—Na1^ii^	111.79 (16)	O10^vi^—Na4—C2—O4	171.2 (3)
C10^i^—Na1—O2—Na1^ii^	119.93 (16)	O1^iii^—Na4—C2—O4	−84.6 (3)
O8^i^—Na1—O2—Na4^iii^	−11.5 (5)	O15—Na4—C2—O4	78.8 (3)
O2^ii^—Na1—O2—Na4^iii^	−97.52 (16)	O3—Na4—C2—O4	−170.6 (4)
O8—Na1—O2—Na4^iii^	109.39 (15)	O2^iii^—Na4—C2—O4	−76.2 (3)
O10—Na1—O2—Na4^iii^	−176.37 (14)	O9^vii^—Na4—C2—O4	17.6 (3)
O9^i^—Na1—O2—Na4^iii^	14.27 (16)	O10^vi^—Na4—C2—C4	64 (2)
O7—Na3—O3—C2	59.7 (4)	O1^iii^—Na4—C2—C4	168 (2)
O4^v^—Na3—O3—C2	164.7 (4)	O15—Na4—C2—C4	−28 (2)
O14—Na3—O3—C2	−44.0 (5)	O3—Na4—C2—C4	82 (2)
O11^vi^—Na3—O3—C2	−111.7 (4)	O2^iii^—Na4—C2—C4	177 (2)
O7—Na3—O3—Na2^iii^	−65.70 (16)	O4—Na4—C2—C4	−107 (2)
O4^v^—Na3—O3—Na2^iii^	39.3 (3)	O9^vii^—Na4—C2—C4	−89 (2)
O14—Na3—O3—Na2^iii^	−169.41 (19)	O10^vi^—Na4—C2—Na2	−135.11 (17)
O11^vi^—Na3—O3—Na2^iii^	122.85 (15)	O1^iii^—Na4—C2—Na2	−30.95 (11)
O7—Na3—O3—Na4	−157.15 (19)	O15—Na4—C2—Na2	132.50 (17)
O4^v^—Na3—O3—Na4	−52.2 (3)	O3—Na4—C2—Na2	−116.9 (3)
O14—Na3—O3—Na4	99.1 (2)	O2^iii^—Na4—C2—Na2	−22.5 (2)
O11^vi^—Na3—O3—Na4	31.4 (2)	O4—Na4—C2—Na2	53.7 (2)
O10^vi^—Na4—O3—C2	164.5 (3)	O9^vii^—Na4—C2—Na2	71.27 (15)
O1^iii^—Na4—O3—C2	−91.0 (3)	O1^iii^—Na2—C2—O3	−20.1 (3)
O15—Na4—O3—C2	70.2 (3)	O1—Na2—C2—O3	78.8 (3)
O2^iii^—Na4—O3—C2	−110.2 (3)	O13—Na2—C2—O3	−114.2 (3)
O4—Na4—O3—C2	−5.2 (3)	O13^iv^—Na2—C2—O3	153.7 (3)
O9^vii^—Na4—O3—C2	11.0 (4)	O3^iii^—Na2—C2—O3	40.3 (4)
O10^vi^—Na4—O3—Na3	9.7 (2)	O4—Na2—C2—O3	−124.8 (5)
O1^iii^—Na4—O3—Na3	114.2 (2)	O1^iii^—Na2—C2—O4	104.7 (3)
O15—Na4—O3—Na3	−84.7 (2)	O1—Na2—C2—O4	−156.4 (3)
O2^iii^—Na4—O3—Na3	94.9 (2)	O13—Na2—C2—O4	10.5 (3)
O4—Na4—O3—Na3	−160.0 (2)	O13^iv^—Na2—C2—O4	−81.5 (3)
O9^vii^—Na4—O3—Na3	−143.8 (2)	O3^iii^—Na2—C2—O4	165.1 (3)
O10^vi^—Na4—O3—Na2^iii^	−85.14 (15)	O1^iii^—Na2—C2—C4	−149.8 (3)
O1^iii^—Na4—O3—Na2^iii^	19.36 (13)	O1—Na2—C2—C4	−50.9 (3)
O15—Na4—O3—Na2^iii^	−179.53 (17)	O13—Na2—C2—C4	116.1 (3)
O2^iii^—Na4—O3—Na2^iii^	0.1 (2)	O13^iv^—Na2—C2—C4	24.0 (3)
O4—Na4—O3—Na2^iii^	105.12 (15)	O3^iii^—Na2—C2—C4	−89.4 (3)
O9^vii^—Na4—O3—Na2^iii^	121.3 (2)	O4—Na2—C2—C4	105.5 (4)
C1^iii^—Na4—O3—Na2^iii^	9.97 (16)	O1^iii^—Na2—C2—Na4	32.96 (12)
C2—Na4—O3—Na2^iii^	110.3 (3)	O1—Na2—C2—Na4	131.83 (17)
O1^iii^—Na2—O4—C2	−73.5 (3)	O13—Na2—C2—Na4	−61.18 (16)
O1—Na2—O4—C2	19.9 (3)	O13^iv^—Na2—C2—Na4	−153.27 (14)
O13—Na2—O4—C2	−170.2 (3)	O3^iii^—Na2—C2—Na4	93.33 (19)
O13^iv^—Na2—O4—C2	107.6 (3)	O4—Na2—C2—Na4	−71.7 (3)
O3^iii^—Na2—O4—C2	−28.0 (5)	O2—C1—C3—C4	178.1 (4)
O1^iii^—Na2—O4—Na3^viii^	148.55 (13)	O1—C1—C3—C4	−3.1 (7)
O1—Na2—O4—Na3^viii^	−118.03 (14)	O2—C1—C3—C8	−4.0 (7)
O13—Na2—O4—Na3^viii^	51.86 (14)	O1—C1—C3—C8	174.9 (4)
O13^iv^—Na2—O4—Na3^viii^	−30.31 (16)	C8—C3—C4—C5	−1.1 (7)
O3^iii^—Na2—O4—Na3^viii^	−166.0 (3)	C1—C3—C4—C5	176.8 (4)
C2—Na2—O4—Na3^viii^	−138.0 (3)	C8—C3—C4—C2	−179.1 (4)
O1^iii^—Na2—O4—Na4	15.07 (13)	C1—C3—C4—C2	−1.1 (7)
O1—Na2—O4—Na4	108.50 (13)	O3—C2—C4—C5	97.5 (5)
O13—Na2—O4—Na4	−81.61 (13)	O4—C2—C4—C5	−84.2 (6)
O13^iv^—Na2—O4—Na4	−163.78 (13)	O3—C2—C4—C3	−84.6 (6)
O3^iii^—Na2—O4—Na4	60.5 (4)	O4—C2—C4—C3	93.7 (5)
C2—Na2—O4—Na4	88.6 (3)	C3—C4—C5—C6	1.1 (8)
O10^vi^—Na4—O4—C2	−13.9 (4)	C2—C4—C5—C6	179.1 (5)
O1^iii^—Na4—O4—C2	92.1 (3)	C3—C4—C5—N1	−178.9 (5)
O15—Na4—O4—C2	−99.0 (3)	C2—C4—C5—N1	−0.8 (7)
O3—Na4—O4—C2	5.2 (2)	O5—N1—C5—C4	−16.7 (9)
O2^iii^—Na4—O4—C2	124.1 (3)	O6—N1—C5—C4	−171.9 (10)
O9^vii^—Na4—O4—C2	−163.6 (3)	O6'—N1—C5—C4	131.8 (11)
C1^iii^—Na4—O4—C2	105.6 (3)	O5—N1—C5—C6	163.4 (6)
O10^vi^—Na4—O4—Na3^viii^	146.4 (3)	O6—N1—C5—C6	8.1 (12)
O1^iii^—Na4—O4—Na3^viii^	−107.6 (2)	O6'—N1—C5—C6	−48.2 (12)
O15—Na4—O4—Na3^viii^	61.3 (2)	C4—C5—C6—C7	−0.2 (9)
O3—Na4—O4—Na3^viii^	165.4 (3)	N1—C5—C6—C7	179.8 (5)
O2^iii^—Na4—O4—Na3^viii^	−75.6 (3)	C5—C6—C7—C8	−0.7 (8)
O9^vii^—Na4—O4—Na3^viii^	−3.3 (2)	C6—C7—C8—C3	0.7 (8)
C1^iii^—Na4—O4—Na3^viii^	−94.1 (2)	C4—C3—C8—C7	0.3 (7)
C2—Na4—O4—Na3^viii^	160.3 (4)	C1—C3—C8—C7	−177.8 (5)
O10^vi^—Na4—O4—Na2	−120.4 (3)	Na1^i^—O8—C9—O7	100.8 (6)
O1^iii^—Na4—O4—Na2	−14.41 (13)	Na1—O8—C9—O7	−101.8 (6)
O15—Na4—O4—Na2	154.48 (18)	Na1^i^—O8—C9—C11	−80.0 (7)
O3—Na4—O4—Na2	−101.40 (15)	Na1—O8—C9—C11	77.5 (6)
O2^iii^—Na4—O4—Na2	17.56 (19)	Na3—O7—C9—O8	177.4 (14)
O9^vii^—Na4—O4—Na2	89.89 (13)	Na3—O7—C9—C11	−2(2)
O3—Na3—O7—C9	−102.8 (17)	Na4^x^—O10—C10—O9	117.5 (8)
O4^v^—Na3—O7—C9	115.6 (17)	Na1—O10—C10—O9	−80.8 (7)
O14—Na3—O7—C9	8.8 (18)	Na4^x^—O10—C10—C12	−63.6 (10)
O11^vi^—Na3—O7—C9	−16 (3)	Na1—O10—C10—C12	98.1 (5)
O2—Na1—O8—C9	32.0 (5)	Na1^i^—O9—C10—O10	69.8 (7)
O8^i^—Na1—O8—C9	−165.3 (5)	Na4^ix^—O9—C10—O10	−86.9 (14)
O2^ii^—Na1—O8—C9	−50.5 (7)	Na1^i^—O9—C10—C12	−109.1 (4)
O10—Na1—O8—C9	−79.7 (5)	Na4^ix^—O9—C10—C12	94.2 (13)
O9^i^—Na1—O8—C9	119.9 (5)	O8—C9—C11—C16	−177.7 (5)
O2—Na1—O8—Na1^i^	−162.62 (15)	O7—C9—C11—C16	1.6 (7)
O8^i^—Na1—O8—Na1^i^	0.0	O8—C9—C11—C12	1.6 (7)
O2^ii^—Na1—O8—Na1^i^	114.8 (3)	O7—C9—C11—C12	−179.1 (5)
O10—Na1—O8—Na1^i^	85.65 (16)	C16—C11—C12—C13	1.8 (8)
O9^i^—Na1—O8—Na1^i^	−74.71 (15)	C9—C11—C12—C13	−177.5 (5)
O2—Na1—O10—C10	−105.2 (5)	C16—C11—C12—C10	−177.7 (5)
O8^i^—Na1—O10—C10	80.0 (5)	C9—C11—C12—C10	3.0 (8)
O2^ii^—Na1—O10—C10	176.1 (5)	O10—C10—C12—C13	88.0 (7)
O8—Na1—O10—C10	−16.8 (4)	O9—C10—C12—C13	−92.9 (7)
O9^i^—Na1—O10—C10	45.6 (7)	O10—C10—C12—C11	−92.5 (6)
O2—Na1—O10—Na4^x^	65.52 (19)	O9—C10—C12—C11	86.5 (7)
O8^i^—Na1—O10—Na4^x^	−109.31 (18)	C11—C12—C13—C14	−0.7 (9)
O2^ii^—Na1—O10—Na4^x^	−13.19 (16)	C10—C12—C13—C14	178.8 (6)
O8—Na1—O10—Na4^x^	154.0 (2)	C11—C12—C13—N2	179.9 (5)
O9^i^—Na1—O10—Na4^x^	−143.7 (4)	C10—C12—C13—N2	−0.7 (9)
O12—N2—O11—Na3^x^	6.4 (14)	O12—N2—C13—C14	7.1 (9)
O1^iii^—Na2—O13—Na2^iv^	162.01 (16)	O11—N2—C13—C14	−171.5 (6)
O1—Na2—O13—Na2^iv^	−72.9 (5)	O12—N2—C13—C12	−173.4 (6)
O13^iv^—Na2—O13—Na2^iv^	0.0	O11—N2—C13—C12	8.0 (9)
O3^iii^—Na2—O13—Na2^iv^	84.68 (16)	C12—C13—C14—C15	−1.2 (10)
O4—Na2—O13—Na2^iv^	−109.64 (15)	N2—C13—C14—C15	178.3 (6)
Na1—O2—C1—O1	99.1 (5)	C13—C14—C15—C16	1.8 (10)
Na1^ii^—O2—C1—O1	−101.6 (5)	C12—C11—C16—C15	−1.2 (9)
Na4^iii^—O2—C1—O1	−4.7 (5)	C9—C11—C16—C15	178.1 (5)
Na1—O2—C1—C3	−82.1 (5)	C14—C15—C16—C11	−0.6 (10)

Symmetry codes: (i) −*x*, −*y*, −*z*+1; (ii) −*x*+1, −*y*, −*z*+1; (iii) −*x*+1, −*y*+1, −*z*+1; (iv) −*x*+2, −*y*+1, −*z*+1; (v) *x*−1, *y*, *z*; (vi) *x*, *y*+1, *z*; (vii) *x*+1, *y*+1, *z*; (viii) *x*+1, *y*, *z*; (ix) *x*−1, *y*−1, *z*; (x) *x*, *y*−1, *z*.

### Hydrogen-bond geometry (Å, °)

**Table:**

*D*—H···*A*	*D*—H	H···*A*	*D*···*A*	*D*—H···*A*
O13—H13A···O7^iii^	0.85	1.94	2.789 (6)	180
O13—H13A···O8^iii^	0.85	2.51	3.049 (5)	123
O13—H13B···O10^vii^	0.85	2.13	2.980 (6)	180
O13—H13B···O9^vii^	0.85	2.63	3.186 (6)	125
O14—H14B···O6^xi^	0.85	1.93	2.782 (13)	179
O14—H14B···O6'^xi^	0.85	1.97	2.722 (15)	147
O14—H14C···O6'^v^	0.85	1.45	2.29 (2)	166
O14—H14C···N1^v^	0.85	2.27	3.078 (10)	160
O14—H14C···O6^v^	0.85	2.55	3.388 (18)	171
O15—H15B···O5	0.85	2.13	2.951 (7)	162
O15—H15C···O9^vii^	0.85	2.29	2.782 (8)	117
O15—H15C···O11^vii^	0.85	2.48	3.302 (8)	163

Symmetry codes: (iii) −*x*+1, −*y*+1, −*z*+1; (vii) *x*+1, *y*+1, *z*; (xi) −*x*+1, −*y*+1, −*z*; (v) *x*−1, *y*, *z*.
